# Exploration of ferroptosis and necroptosis-related genes and potential molecular mechanisms in psoriasis and atherosclerosis

**DOI:** 10.3389/fimmu.2024.1372303

**Published:** 2024-07-12

**Authors:** Jilin Fan, Tingting Zhu, Xiaoling Tian, Sijia Liu, Shi-Liang Zhang

**Affiliations:** ^1^ The First Clinical Medical College, Shandong University of Traditional Chinese Medicine, Jinan, China; ^2^ Department of Neurosurgery Ward 5, Binzhou Medical University Hospital, Binzhou, Shandong, China; ^3^ Cardiovascular Department, Affiliated Hospital of Shandong University of Traditional Chinese Medicine, Jinan, Shandong, China

**Keywords:** psoriasis, atherosclerosis, ferroptosis, necroptosis, ceRNA network, immune infiltration

## Abstract

**Objective:**

Ferroptosis and necroptosis are two recently identified forms of non-apoptotic cell death. Their dysregulation plays a critical role in the development and progression of Psoriasis (PsD) and Atherosclerosis (AS). This study explores shared Ferroptosis and necroptosis-related genes and elucidates their molecular mechanisms in PsD and AS through the analysis of public databases.

**Methods:**

Data sets for PsD (GSE30999) and AS (GSE28829) were retrieved from the GEO database. Differential gene expression (DEG) and weighted gene co-expression network analysis (WGCNA) were performed. Machine learning algorithms identified candidate biomarkers, whose diagnostic values were assessed using Receiver Operating Characteristic (ROC) curve analysis. Additionally, the expression levels of these biomarkers in cell models of AS and PsD were quantitatively measured using Western Blot (WB) and real-time quantitative PCR (RT-qPCR). Furthermore, CIBERSORT evaluated immune cell infiltration in PsD and AS tissues, highlighting the correlation between characteristic genes and immune cells. Predictive analysis for candidate drugs targeting characteristic genes was conducted using the DGIdb database, and an lncRNA-miRNA-mRNA network related to these genes was constructed.

**Results:**

We identified 44 differentially expressed ferroptosis-related genes (DE-FRGs) and 30 differentially expressed necroptosis-related genes (DE-NRGs). GO and KEGG enrichment analyses revealed significant enrichment of these genes in immune-related and inflammatory pathways, especially in NOD-like receptor and TNF signaling pathways. Two ferroptosis-related genes (NAMPT, ZFP36) and eight necroptosis-related genes (C7, CARD6, CASP1, CTSD, HMOX1, NOD2, PYCARD, TNFRSF21) showed high sensitivity and specificity in ROC curve analysis. These findings were corroborated in external validation datasets and cell models. Immune infiltration analysis revealed increased levels of T cells gamma delta, Macrophages M0, and Macrophages M2 in PsD and AS samples. Additionally, we identified 43 drugs targeting 5 characteristic genes. Notably, the XIST-miR-93–5p-ZFP36/HMOX1 and NEAT1-miR-93–5p-ZFP36/HMOX1 pathways have been identified as promising RNA regulatory pathways in AS and PsD.

**Conclusion:**

The two ferroptosis-related genes (NAMPT, ZFP36) and eight necroptosis-related genes (C7, CARD6, CASP1, CTSD, HMOX1, NOD2, PYCARD, TNFRSF21) are potential key biomarkers for PsD and AS. These genes significantly influence the pathogenesis of PsD and AS by modulating macrophage activity, participating in immune regulation, and mediating inflammatory responses.

## 1 Introduction

Psoriasis (PsD) is a chronic, immune-mediated inflammatory skin condition with a genetic predisposition, affecting an estimated 2%-5% of the global population (1). It primarily affects the skin and joints, but also significantly influences the cardiovascular system through systemic inflammation (2). This inflammation not only exacerbates PsD symptoms but also affects the vascular system and contributes to the formation and progression of Atherosclerosis (AS) plaques (3). Emerging evidence identifies PsD as an independent risk factor for AS, with patients experiencing PsD for over eight years facing a heightened risk of developing AS (4, 5). Although PsD is recognized as an independent risk factor for AS, the pathogenic mechanisms of their coexistence are not fully understood.

The relationship between PsD and AS is complex, with existing research highlighting shared immune mechanisms involving the IL-12/Th1 and IL-23/Th17 pathways. Differentiated Th1 cells produce TNF-α and IFN-γ, which promote plaque growth, while Th17 effector cytokines contribute to neovascularization and bleeding within plaques, thus enhancing their fragility (6). Furthermore, a decrease in Treg cells and their functions leads to increased Th1 and Th17 proliferation. This is accompanied by reductions in TGF-β and IL-10, both known for their anti-inflammatory properties and roles in AS protection (7). In PsD, activated Th17 cells produce pro-inflammatory cytokines IL-17A-F, IL-22, IL-21, and TNF-α. TNF-α stimulates IL-23 formation, thereby enhancing memory T cell synthesis of IL-17 and IFN-γ, which perpetuates skin inflammation (8, 9). These insights indicate that immune-mediated inflammation plays a crucial role in the dynamics between PsD and AS, creating a detrimental cycle that significantly impacts patients. Therefore, understanding the pathogenesis of PsD and AS and identifying shared therapeutic targets is of paramount importance.

Moreover, ferroptosis and necroptosis are distinct from autophagy, pyroptosis, and apoptosis, and they play a key role in AS and PsD (10, 11). For instance, studies have shown a significant reduction in GPX4 in PsD lesions compared to healthy uninvolved skin, along with a marked increase in Nrf2 downstream targets (12). Li et al. observed elevated levels of lipid ROS and divalent iron in the epidermis of PsD patients and confirmed the activation of ferroptosis in PsD patients and mouse models using transmission electron microscopy (13). Similarly, Fer-1 has been shown to mitigate lipid peroxidation and endothelial dysfunction in mouse aortic endothelial cells by upregulating SLC7A11 and GPX4 expression, thereby delaying the pathological progression of AS (14). Additionally, studies indicate that knocking out ApoA1 leads to increased formation of necrotic cores in atherosclerotic plaques and elevated phosphorylation levels of necroptotic mediators RIPK3 and MLKL (15). In PsD, increased expression of necroptotic molecules RIPK1, RIPK3, and MLKL has been noted, particularly in keratin-forming cells of the epidermis (16). These findings underscore the significance of ferroptosis and necroptosis in both PsD and AS. However, the specific molecular mechanisms driving this relationship remain unclear. Increasing evidence suggests that ferroptosis and necroptosis are not independent processes, but rather interact extensively. For instance, mitochondrial ROS are essential for the pathogenesis of both ferroptosis and necroptosis, facilitating the autophosphorylation of RIPK1, which subsequently recruits RIPK3 to form functional necrosomes (17). Additionally, studies have shown that overexpression of GPX4 can reduce mitochondrial ROS levels, thereby preventing both ferroptosis and necroptosis (18). Furthermore, knocking out ACSL4 inhibits ferroptosis in iron-sensitive mouse and human cells, but increases their sensitivity to necroptosis (19). Recent research indicates that the release of damage-associated molecular patterns (DAMPs) from plasma membrane pores may be a common feature of both ferroptosis and necroptosis, suggesting that DAMPs released during ferroptosis may promote necroptosis. These findings highlight the interconnected nature of ferroptosis and necroptosis (20). However, most studies to date have focused on either ferroptosis or necroptosis as singular cell death mechanisms in relation to disease, with less attention given to their combined pathogenic mechanisms in diseases. Therefore, further exploration of the shared genetic traits and potential molecular mechanisms of ferroptosis and necroptosis in PsD and AS holds broad prospects for the diagnosis and treatment of these comorbid conditions.

**Figure 1:**
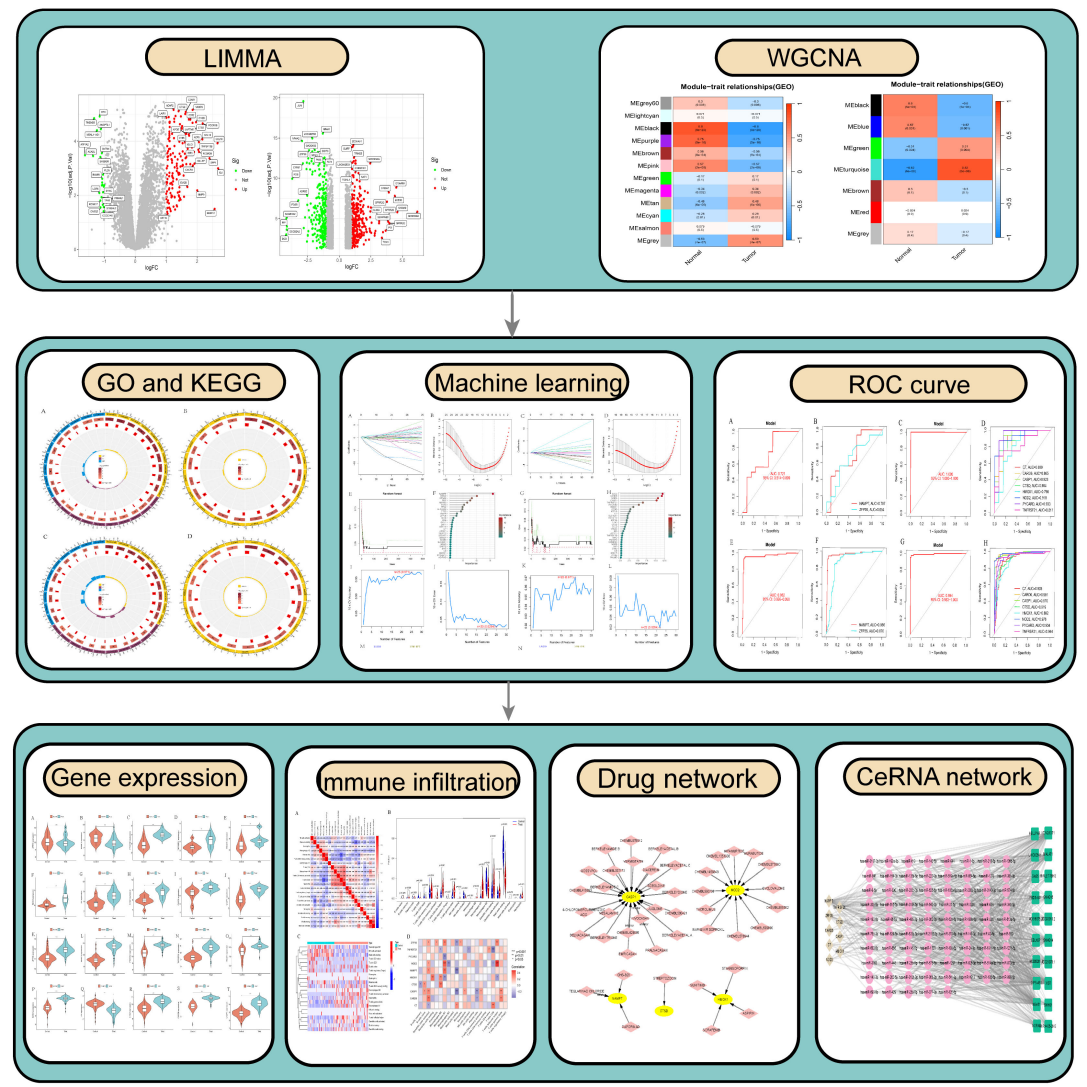
Flow chart.

With the advancement of microarray high-throughput squencing technology in recent years, microarrays have become a pioneering method for identifying genes associated with ferroptosis and necroptosis in PsD and AS, facilitating early diagnosis and treatment (21). Integrating bioinformatics approaches enables the precise identification of potential molecular biomarkers of ferroptosis and necroptosis in PsD and AS, thereby enhancing our understanding of their biological mechanisms and pinpointing exact molecular targets for therapy. Accordingly, this study utilizes the limma package and Weighted Gene Co-expression Network Analysis (WGCNA) to pinpoint differentially expressed genes in PsD and AS. We then employ machine learning techniques to identify characteristic genes of ferroptosis and necroptosis, and to explore their biological processes and immune infiltration mechanisms. These characteristic genes could prove to be crucial regulators and potential therapeutic targets in PsD and AS (**Figure 1**).

## 2 Materials and methods

### 2.1 Dataset acquisition

Expression data for atherosclerosis (AS), psoriatic dermatitis (PsD), and normal samples were obtained from the GEO database. The selection criteria included: 1) Usage of the GPL570 [HG-U133_Plus_2] Affymetrix Human Genome U133 Plus 2.0 Array for all datasets; 2) Inclusion of only human subjects; 3) A minimum of 20 samples per study; 4) Collection of samples from either skin or arterial tissues. Datasets GSE28829 and GSE30999 were selected as the training set for this study, owing to their comprehensive and reliable data extensively cited in numerous publications (22, 23). Gene expression datasets for GSE28829 and GSE30999 were retrieved from the GEO database utilizing the Affymetrix GPL570 platform. The GSE28829 dataset includes 16 advanced atherosclerotic plaques and 13 early atherosclerotic plaques, while the GSE30999 dataset contains 85 paired samples from psoriasis patients, featuring both lesional skin (LS) and adjacent normal tissues (NL).Additionally, datasets GSE100927 and GSE14905, noted for their extensive citation and verification through *in vitro* studies, were selected as the validation sets for this research (24, 25).

### 2.2 Differential expression analysis

We employed the R package “limma” (version 3.40.6), which uses a generalized linear model approach to identify differentially expressed genes (DEGs) between the diseased and normal groups within the GSE28829 and GSE30999 datasets (26). The selection criteria were a P.Value < 0.05 and an absolute log2 fold change (log2FC) of ≥ 0.5. Volcano plots for the DEGs were generated using the “ggplot2” package (27). DEGs common to both datasets were selected as candidate genes for further analysis.

### 2.3 WGCNA analysis

WGCNA, a systems bioinformatics technique, efficiently groups highly correlated genes into modules and analyzes correlations between these modules and phenotypes (28). We conducted “WGCNA” on the GSE28829 and GSE30999 datasets using the “WGCNA” package in R. The analysis began with sample clustering, excluding genes with an average expression below 0.5. The soft-thresholding power β was determined using the pickSoftThreshold function in R. Hierarchical clustering analysis was performed with a minimum module size of 60 and a merge height cutoff of 0.25. Finally, we calculated the gene significance and module membership for disease-associated modules. Notably, the grey module was categorized as a collection of genes not assigned to any module. We selected modules for further analysis based on a Pearson correlation coefficient |r| > 0.5 and P < 0.05.

### 2.4 Identification of differentially expressed genes related to ferroptosis and necroptosis

Genes associated with ferroptosis were sourced from the FerrDb and GeneCards databases, while those pertaining to necroptosis were obtained from the GeneCards and Harmonizome databases. The differentially expressed genes (DEGs) identified in Section 1.2 and module genes from Section 1.3 were merged. This combined set was then cross-referenced with the ferroptosis and necroptosis gene sets to pinpoint candidate genes for further analysis.

### 2.5 Functional enrichment analysis

To elucidate the biological functions and pathways related to DE-FRGs and DE-NRGs in PsD and AS, gene ontology (GO) and Kyoto Encyclopedia of Genes and Genomes (KEGG) enrichment analyses were conducted using the DAVID database (29). GO analysis examined the genes’ biological processes (BP), cellular components (CC), and molecular functions (MF), while KEGG analysis assessed the pathways in which these genes are predominantly involved. Significance was indicated by a P-value < 0.05, highlighting significant enrichment of DE-FRGs and DE-NRGs in various GO and KEGG categories.

### 2.6 Immune cell infiltration analysis

CIBERSORT, a tool for deconvoluting human immune cell subtype expression matrices using linear support vector regression, determines the proportions of different immune cell types based on known gene expression profiles. This analysis helps delineate the immune cell composition within the immune microenvironment of PsD and AS, identifying key immune cells involved in the development and progression of these diseases (30). The “CIBERSORT” package in R was utilized for analyzing immune infiltration in control samples versus PsD and AS samples. Bar graphs depicted the proportions of various immune cell types, while violin plots contrasted the levels of immune cell infiltration between PsD and control groups, and between AS and control groups. Additionally, this study demonstrated the correlation between the expression levels of hub genes and the degree of infiltration of various immune cell types in the samples.

### 2.7 Identification of key DE-FRGs and DE-NRGs

To pinpoint key DE-FRGs and DE-NRGs linked to PsD and AS, three machine learning algorithms were employed: LASSO, SVM-RFE, and RF. The LASSO algorithm was used to identify genetic biomarkers of PsD and AS by contrasting DE-FRGs and DE-NRGs against normal samples (31). SVM, a supervised machine learning method for data classification, assessed model performance through the average false-positive rate obtained from 10-fold cross-validation (32). The “randomForest” package in the RF algorithm assessed the feature importance of each gene, deeming those with an importance value above 2 as potential biomarkers for further investigation (33). Ultimately, the genes identified by LASSO, SVM-RFE, and RF were intersected to aid in the diagnosis of PsD and AS.

### 2.8 Identification and analysis of hub genes

The diagnostic value of hub genes in PsD and AS was determined using the pROC R package, which constructed Receiver Operating Characteristic (ROC) curves and calculated the area under the curve (AUC) for each gene. An AUC > 0.5 was considered to have diagnostic value (34). The expression levels of these hub genes in PsD and AS were confirmed using the training sets (GSE28829 and GSE30999) and validated in the validation sets (GSE100927 and GSE14905). Additionally, the corrplot R package generated correlation heatmaps, illustrating the relationships between immune cells and candidate diagnostic genes.

### 2.9 Prediction of candidate drugs for hub genes

Drugs associated with the hub genes were identified using the Drug-Gene Interaction database (DGIdb) (35). The DGIdb database facilitated the exploration of potential drugs or compounds that interact with DE-FRGs and DE-NRGs. Subsequently, a drug-gene interaction network was visualized using Cytoscape software, offering an in-depth view of the interactions between these hub genes and potential therapeutic agents.

### 2.10 Construction of the CeRNA network

MiRNAs targeting the hub genes were identified using the miRanda, miRDB, and TargetScan databases, while lncRNAs potentially targeting these miRNAs were sourced from the starBase database. A ceRNA network involving lncRNA-miRNA-mRNA interactions was then constructed using Cytoscape software, providing a comprehensive framework for understanding these interactions.

### 2.11 Cell culture and treatment

Human epidermal keratinocytes (HaCaT) and human umbilical vein endothelial cells (HUVEC) cell lines were obtained from Wuhan Pricella Biotechnology Co., Ltd. (Wuhan, China). HaCaT cells were cultured in DMEM containing 10% serum, 1% penicillin, and 1% streptomycin, while HUVEC cells were maintained in complete endothelial cell culture medium (ZQ1304, Zhongqiaoxinzhou Biotech). Both cell types were incubated at 37°C in a 5% CO_2_ incubator and passaged upon reaching approximately 80% confluence. As previously described, HaCaT cells were treated with a concentration of 10ng/ml of M5 (TNF-α, IL-17A, IL-22, IL-1α, and oncostatin M) for approximately 24 hours to induce a PsD inflammation cell model (36). Additionally, HUVEC were exposed to 100 μg/mL ox-LDL for 24 hours to simulate an *in vitro* AS injury model (37).

### 2.12 Western blotting

Total protein was extracted from HaCaT and HUVEC cells using RIPA lysis buffer containing protease and phosphatase inhibitors. Protein concentration was estimated using the BCA assay. Samples were separated on SDS-polyacrylamide gel electrophoresis (SDS-PAGE) and then transferred to PVDF membranes. To block the membranes, 10% non-fat milk was used for two hours, followed by overnight incubation at 4°C with primary antibodies diluted appropriately (anti-NAMPT, ZFP36, CTSD, HMOX1, CASP1, and PYCARD antibodies). Membranes were then incubated with HRP-conjugated secondary antibodies at room temperature for one hour. Detection was performed using a highly sensitive multi-function imager (GE, USA), and images were analyzed using Image J software.

### 2.13 Real-time quantitative PCR analysis

Total RNA was isolated from HaCaT and HUVEC cells using TRIzol reagent. The extracted RNA was reverse-transcribed into complementary DNA using a reverse transcription kit. qRT-PCR analysis was conducted using a SYBR Green reagent kit and a real-time fluorescence PCR instrument. The levels of target mRNA were normalized to the levels of GAPDH mRNA (internal control). Primers used in the qRT-PCR analysis are listed in **Table 1**.

**Table 1:** PCR primer sequences.

RNA	Forward	Reverse
NAMPT	CCTGATTCTGGAAACCCTCTTGAC	AGATAAGGTGGCAGCAACTTGTAAC
CTSD	ACCACAAGTACAACAGCGACAAG	GGCAGGGCACCGACACAG
HMOX1	GCCAGTGCCACCAAGTTCAAG	GATGTTGAGCAGGAACGCAGTC
CASP1	GGTGCTGAACAAGGAAGAGATGG	TCGGAATAACGGAGTCAATCAAAGC
PYCARD	CGTTGAGTGGCTGCTGGATG	CAGGCTGGTGTGAAACTGAAGAG
ZFP36	GCTGCCACTTCATCCACAACC	GCCTGGTGGTGGTGGTGAG
GAPDH	CAGGAGGCATTGCTGATGAT	GAAGGCTGGGGCTCATTT

### 2.14 Statistical analysis

All data processing and analyses in this study were conducted using GraphPad Prism and R software (version 4.1.0). For comparisons between two groups involving continuous variables, the Student’s t-test was used for normally distributed data, with results presented as mean ± SD. ROC curve analyses were performed using the “pROC” package in R, assessing diagnostic performance by calculating the area under the curve (AUC). Correlations between different genes were calculated using Spearman’s rank correlation analysis. All statistical p-values were two-sided, with p < 0.05 considered statistically significant.

## 3 Results

### 3.1 WGCNA identified key modules in psoriasis and atherosclerosis

**Figure 2:**
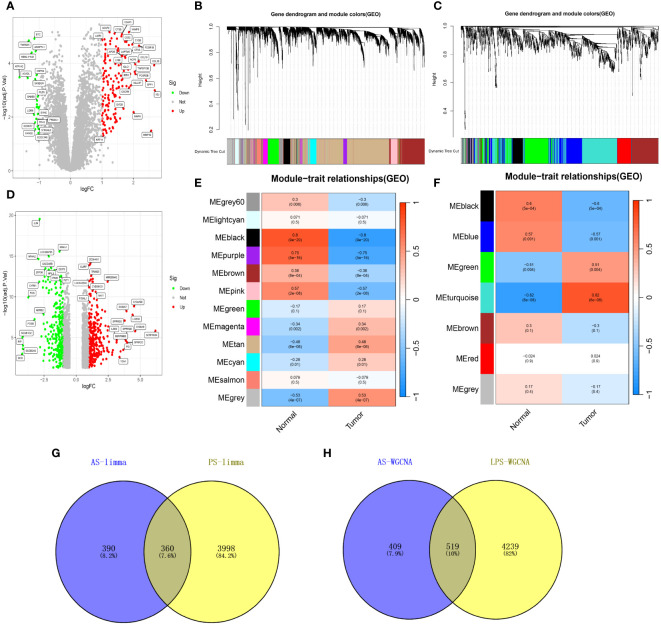
Shared genes in AS and PsD. **(A)** Volcano plot of DEGs in AS. **(B, C)** Hierarchical clustering of genes into different modules (each color represents a module). **(D)** Volcano plot of DEGs in PsD. **(E)** Heatmap of module-trait gene relationships in PsD. **(F)** Heatmap of module-trait gene relationships in AS. **(G)** Venn diagram of common DEGs in AS and PsD. **(H)** Venn diagram of genes in related modules in AS and PsD.

Analysis of datasets GSE28829 and GSE30999 identified 750 and 4358 DEGs, respectively (**Figures 2A, D**), with 360 DEGs common between the two (**Figure 2G**). WGCNA revealed 18 relevant modules in GSE30999 and 7 in GSE28829, each represented by a unique color. Module-trait relationship heatmaps were generated to assess each module’s disease association (**Figures 2B, C, E, F**). The “blue” (|r|=0.57, p=0.001), “black” (|r|=0.6, p=5e-04), “green” (|r|=0.51, p=0.004), and “turquoise” (|r|=0.82, p=6e-08) modules were highly correlated with AS, comprising 928 genes. The “blue” (|r|=0.91, p=4e-66), “brown” (|r|=0.71, p=4e-27), “cyan” (|r|=0.69, p=2e-25), “magenta” (|r|=0.6, p=4e-18), “pink” (|r|=0.53, p=9e-14), and “yellow” (|r|=0.61, p=6e-19) modules were strongly linked with PsD, encompassing 4758 genes. In total, 519 genes were shared across modules associated with both conditions (**Figure 2H**). By integrating the 360 shared DEGs detected by limma with the 519 from WGCNA and eliminating duplicates, a total of 879 shared genes were isolated for further analysis.

### 3.2 Selection of DE-FRGs and DE-NRGs

**Figure 3:**
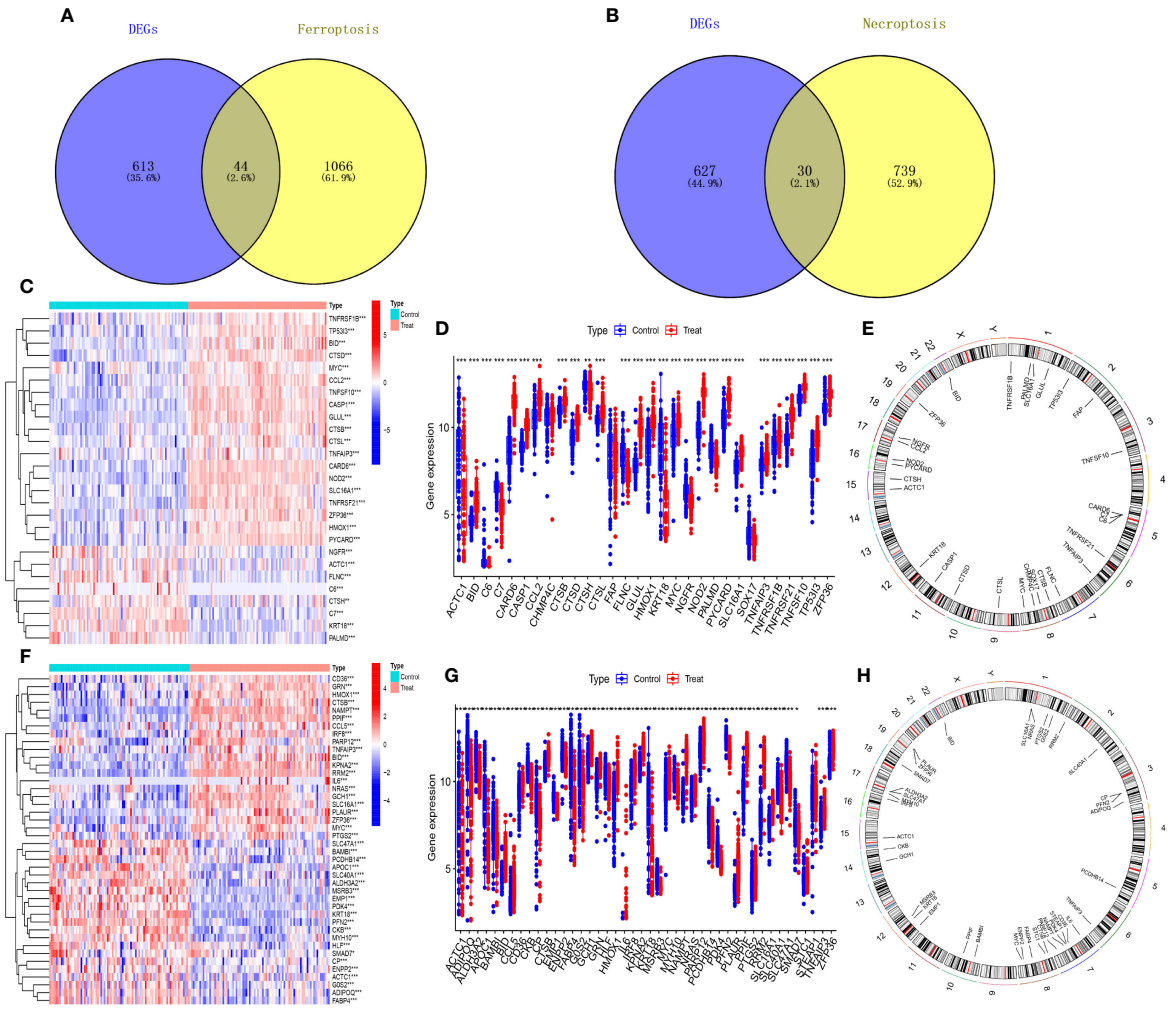
Identification of DE-FRGs and DE-NRGs. **(A)** Venn diagram of shared genes intersecting with ferroptosis-related genes. **(B)** Venn diagram of shared genes intersecting with necroptosis-related genes. **(C, F)** Heatmaps of expression patterns for DE-NRGs and DE-FRGs, respectively. **(D, G)** Bar scatter plots showing expression levels of DE-NRGs and DE-FRGs. **(E, H)** Chromosomal positions of DE-NRGs and DE-FRGs. *P<0.05, **P<0.01, ***P<0.001.

The 879 shared genes were cross-referenced with iron death and necroptotic apoptosis gene sets, yielding 44 DE-FRGs and 30 DE-NRGs (**Figures 3A, B**). The differential expression of these genes is depicted in **Figures 3C, F**, while their expression levels are shown in bar scatter plots (**Figures 3D, G**). The chromosomal locations of the 44 DE-FRGs and 30 DE-NRGs are detailed in **Figures 3E, H**.

### 3.3 GO and KEGG enrichment analyses were conducted to identify biological processes and signaling pathways associated with differentially expressed genes

**Figure 4:**
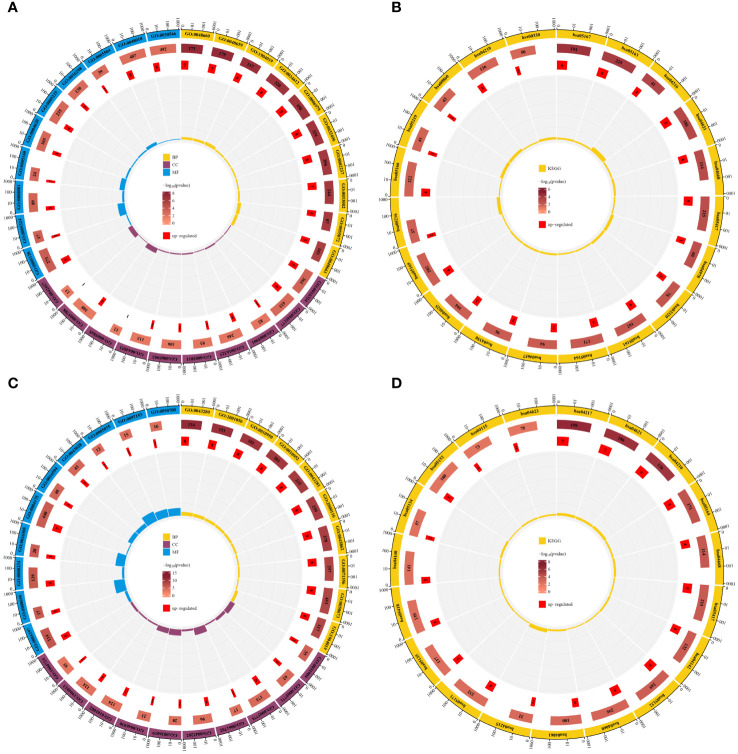
Enrichment analysis of DE-FRGs and DE-NRGs. **(A)** GO function enrichment analysis for DE-FRGs. **(B)** KEGG enrichment analysis for DE-FRGs. **(C)** GO function enrichment analysis for DE-NRGs. **(D)** KEGG enrichment analysis for DE-NRGs.

To elucidate the biological processes and pathways of shared differentially expressed genes, we conducted GO function and KEGG pathway enrichment analyses on the 44 DE-FRGs and 30 DE-NRGs using the DAVID online tool. The GO function enrichment analysis showed that DE-FRGs are predominantly enriched in regulation of smooth muscle cell proliferation, epithelial cell apoptotic process, response to oxidative stress, presence of lipid droplets, endoplasmic reticulum lumen, cytokine receptor binding, transforming growth factor beta receptor binding, and cytokine activity. Conversely, DE-NRGs are mainly enriched in positive regulation of endopeptidase activity, cellular response to tumor necrosis factor, inflammasome complex, pore complex, peptidase activator activity in the apoptotic process, and death receptor activity. The KEGG enrichment analysis indicated significant enrichment of DE-FRGs in pathways such as Ferroptosis, NOD-like receptor signaling pathway, TNF signaling pathway, PPAR signaling pathway, and IL-17 signaling pathway. Conversely, DE-NRGs showed significant enrichment in Necroptosis, NOD-like receptor signaling pathway, TNF signaling pathway, and p53 signaling pathway. These findings highlight the crucial role of inflammatory responses in both PsD and AS, with extracellular immunomediators such as cytokines and pathways like NOD-like receptor signaling and TNF signaling pathways playing central roles (**Figure 4**).

### 3.4 Potential biomarkers for atherosclerosis and psoriasis were screened using three machine learning methods

**Figure 5:**
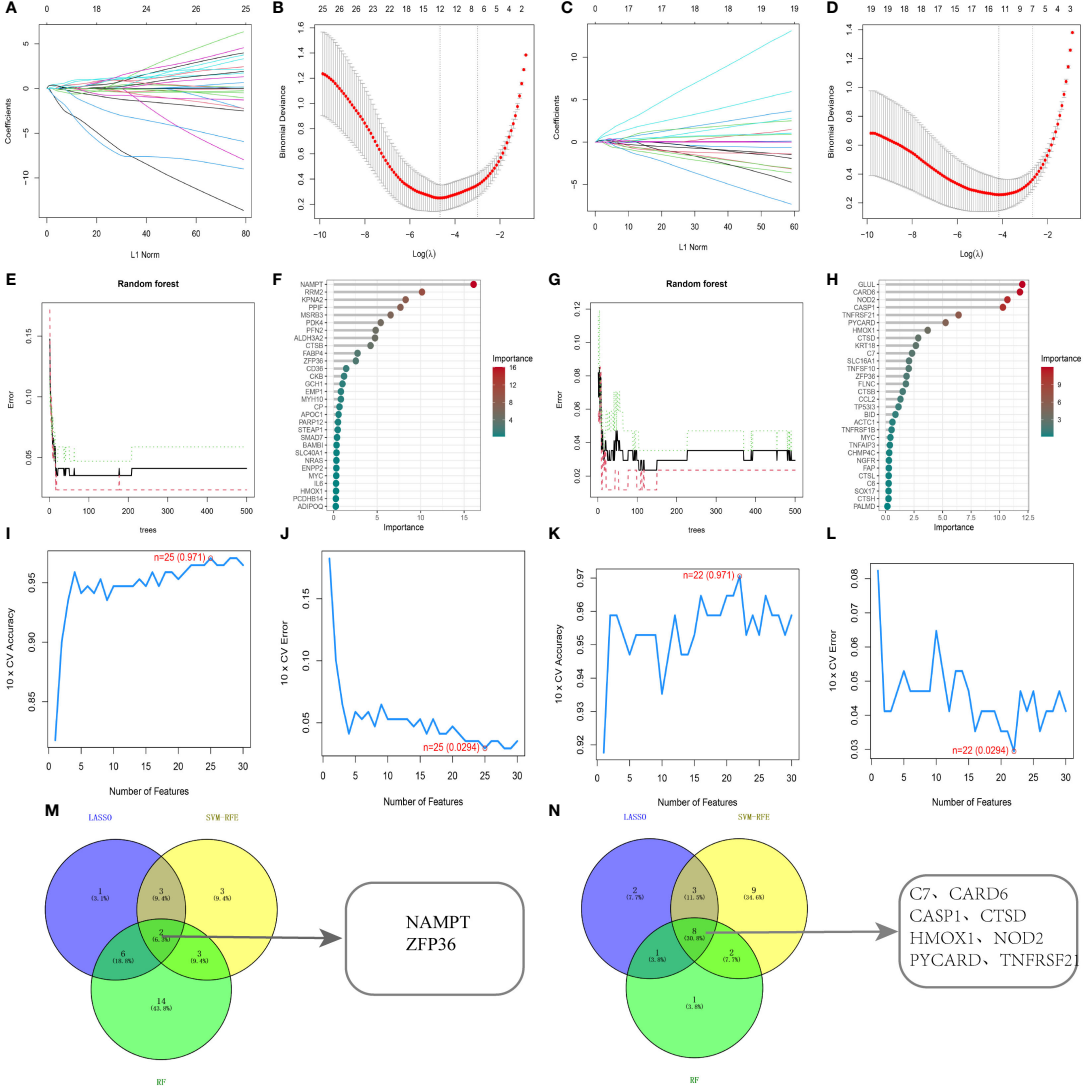
Identification using machine learning algorithms. **(A, B)** LASSO regression for selecting ferroptosis characteristic genes, with the optimal gene count (n=12) at the curve’s lowest point. **(C, D)** LASSO regression for selecting necroptosis characteristic genes, with the optimal gene count (n=14) at the curve’s lowest point. **(E, F)** Random forest algorithm for ferroptosis characteristic gene selection. **(G, H)** Random forest algorithm for necroptosis characteristic gene selection. **(I, J)** SVM-RFE algorithm for ferroptosis characteristic gene selection. **(K, L)** SVM-RFE algorithm for necroptosis characteristic gene selection. **(M, N)** Common genes identified by the three algorithms. .

To determine diagnostic biomarkers for ferroptosis and necroptosis in PsD and AS, we utilized LASSO, SVM-RFE, and RF algorithms for feature gene selection. The LASSO algorithm identified 12 ferroptosis-related genes and 14 necroptosis-related genes (**Figures 5A–D**). The RF algorithm pinpointed 11 genes associated with iron death and 12 related to necroptosis (**Figures 5E–H**). Meanwhile, the SVM-RFE algorithm selected 25 ferroptosis-related genes and 22 necroptosis-related genes (**Figures 5I–L**). Using these algorithms, we identified 2 ferroptosis-related genes (NAMPT, ZFP36) and 8 necroptosis-related genes (C7, CARD6, CASP1, CTSD, HMOX1, NOD2, PYCARD, TNFRSF21) (**Figures 5M, N**) **Table 2**.

**Table 2:** Characteristic gene information.

Genes	Description	Function	logFC	P.Val	PMID
NAMPT	Nicotinamide phosphoribosyltransferase	CatalyticActivity and EnzymeRegulation	0.501	0.02	8289818
CTSD	Cathepsin D heavy chain	CatalyticActivity and Biochemistry	0.81	1.64E-05	16685649
HMOX1	Heme oxygenase 1	CatalyticActivity、Induction and BiophysicochemicalProperties	1.03	0.003	10631150
CASP1	Caspase-1 subunit p10	CatalyticActivity、Induction and EnzymeRegulation	0.92	1.25E-06	1373520
PYCARD	Apoptosis-associated speck-like protein containing a CARD	Induction	1.1	2.21E-07	16964285
ZFP36	mRNA decay activator protein ZFP36	Induction and Biochemistry	0.51	0.02	20221403

### 3.5 Key genes were validated using the GEO database and further confirmed through cellular experiments

**Figure 6:**
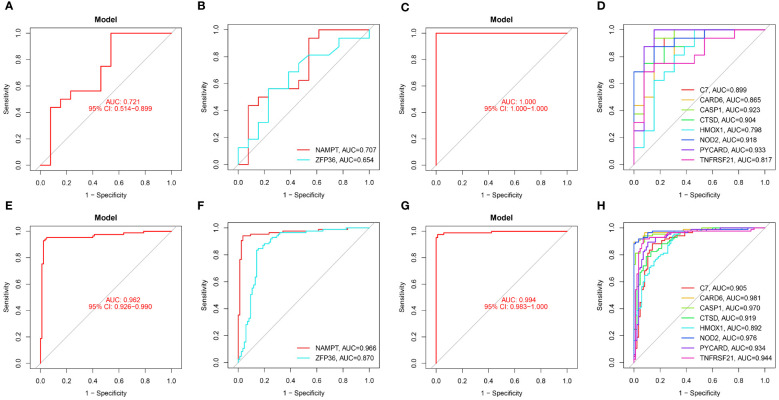
Diagnostic performance of candidate genes in the validation sets. **(A, B)** ROC curve analysis of the 2 ferroptosis genes in GSE28829. **(C, D)** ROC curve analysis of the 8 necroptosis genes in GSE28829. **(E, F)** ROC curve analysis of the 2 ferroptosis genes in GSE30999. **(G, H)** ROC curve analysis of the 8 necroptosis genes in GSE30999.

**Figure 7:**
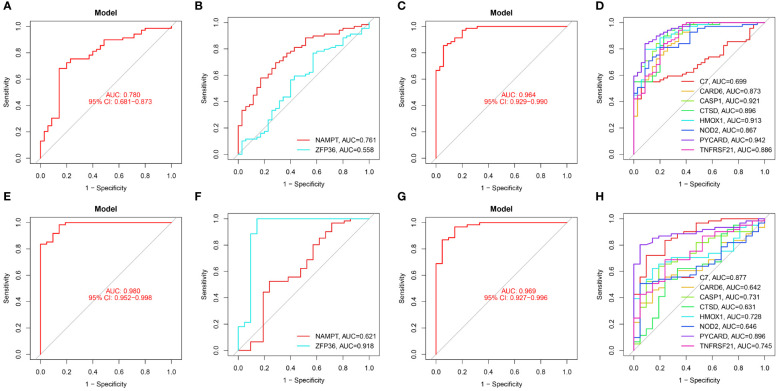
Diagnostic performance of candidate genes in the validation set. **(A, B)** ROC curve results for 2 ferroptosis genes in GSE100927. **(C, D)** ROC curve results for 8 necroptotic apoptosis genes in GSE100927. **(E, F)** ROC curve results for 2 ferroptosis genes in GSE14905. **(G, H)** ROC curve results for 8 necroptotic apoptosis genes in GSE14905.

In our study, we used ROC curves to assess the diagnostic predictive values of hub genes in different datasets (**Figures 6**, **7**). In the GSE28829 dataset, the following genes exhibited AUC values greater than 0.6: ZFP36 (AUC=0.707), NAMPT (AUC=0.654), C7 (AUC=0.889), CARD6 (AUC=0.865), CASP1 (AUC=0.923), CTSD (AUC=0.904), HMOX1 (AUC=0.798), NOD2 (AUC=0.918), PYCARD (AUC=0.933), TNFRSF21 (AUC=0.817). Similarly, in the GSE30999 dataset, ZFP36 (AUC=0.870), NAMPT (AUC=0.966), C7 (AUC=0.905), CARD6 (AUC=0.981), CASP1 (AUC=0.970), CTSD (AUC=0.919), HMOX1 (AUC=0.892), NOD2 (AUC=0.976), PYCARD (AUC=0.934), TNFRSF21 (AUC=0.944) also demonstrated AUC values greater than 0.6, indicating their potential as reliable diagnostic markers for AS and PsD.

In the validation sets, different cohorts also demonstrated good predictive efficacy. In the AS validation set (GSE100927), the AUC values for ZFP36, NAMPT, C7, CARD6, CASP1, CTSD, HMOX1, NOD2, PYCARD, and TNFRSF21 were 0.558, 0.761, 0.699, 0.873, 0.921, 0.896, 0.913, 0.867, 0.942, and 0.886, respectively. In the PsD validation set (GSE14905), the AUC values were 0.918, 0.621, 0.877, 0.642, 0.731, 0.631, 0.728, 0.646, 0.896, and 0.745. These results further suggest that these ten genes could serve as common diagnostic markers for AS and PsD.

**Figure 8:**
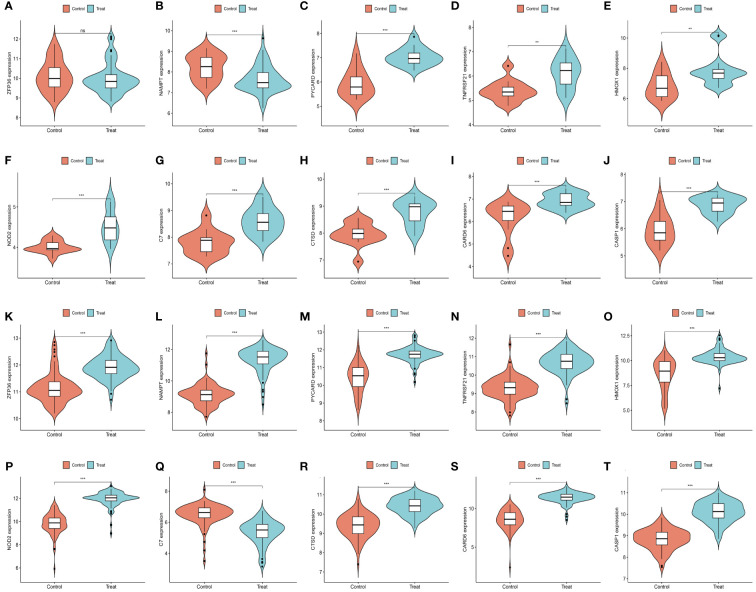
Expression levels of feature genes in GSE28829 and GSE30999. **(A–J)** Expression patterns of feature genes in GSE28829. **(K–T)** Expression patterns of feature genes in GSE30999. **P<0.01, ***P<0.001. ns, No statistical.

**Figure 9:**
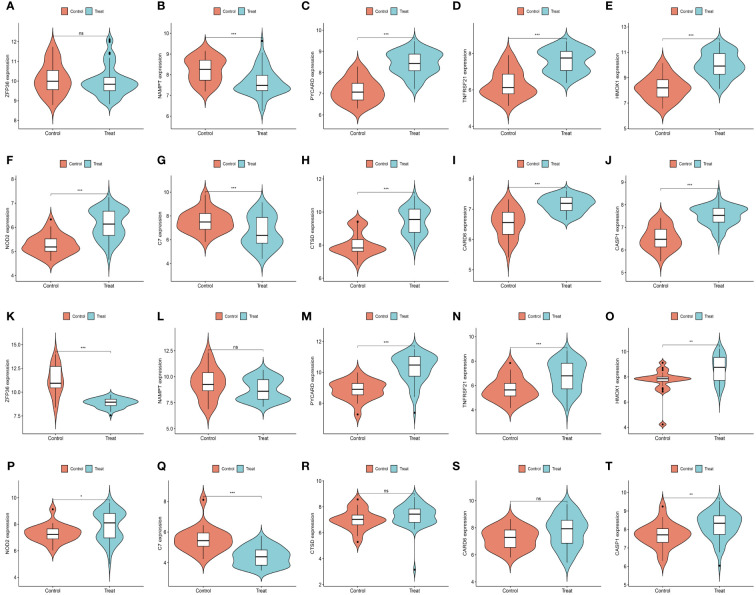
Expression levels of feature genes in GSE100927 and GSE14905. **(A–J)** Expression patterns of feature genes in GSE100927. **(K–T)** Expression patterns of feature genes in GSE14905. *P<0.05, **P<0.01, ***P<0.001. ns, No statistical.

**Figure 10:**
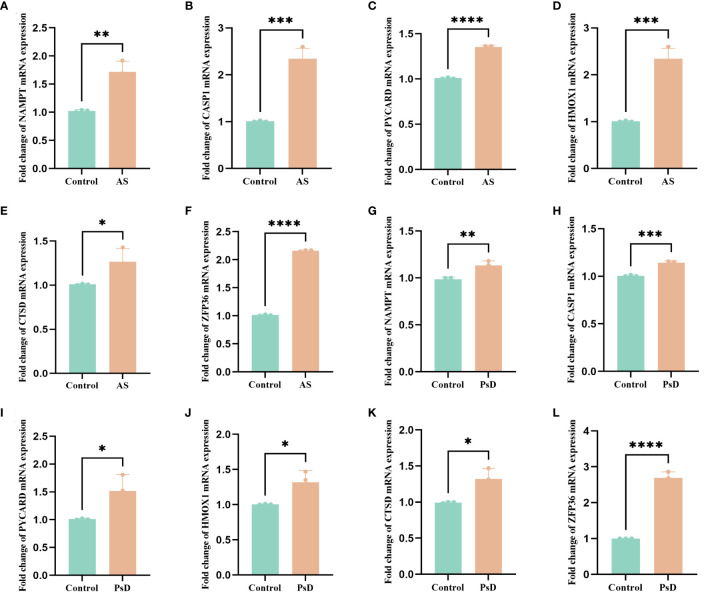
RT-qPCR results of six key genes in as and PsD samples. **(A–F)** Experimental validation of key gene expression in AS. **(G–L)** Experimental validation of key gene expression in PsD samples. Relative mRNA expression of NAMPT, CASP1, PYCARD, HMOX1, CTSD and ZFP36. (*p< 0.05, **p < 0.01, ***p<0.001, ****p<0.0001).

**Figure 11:**
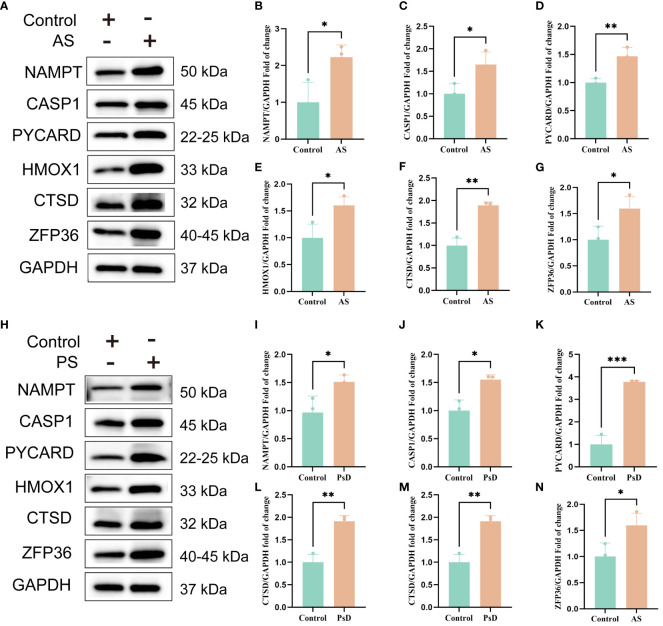
WB results of six key genes in AS and PsD samples. **(A, H)** Western blot analysis of NAMPT, CASP1, PYCARD, HMOX1, CTSD and ZFP36 protein levels in As and PsD samples. **(B–G, I–N)** The ratio of NAMPT, CASP1, PYCARD, HMOX1, CTSD and ZFP36 to GAPDH was quantitatively analyzed by ImageJ. (*p< 0.05, **p < 0.01, ***P<0.001).

We further demonstrated that the expression levels of two ferroptosis genes and eight necroptotic apoptosis genes in the training sets GSE28829 and GSE30999 (**Figures 8A–J**) showed significant differences between the AS group and the normal group, except for ZFP36. These genes also exhibited significant expression differences between the psoriatic disease (PsD) group and the normal group (**Figures 8K–T**). Additionally, as depicted in **Figures 9A–J**, the expression levels of the characteristic genes in GSE100927 were consistent with those in the training sets. Similarly, their expression levels in GSE14905 matched those in the training sets (**Figures 9K–T**). Additionally, RT-qPCR results confirmed that, compared to the control group, the mRNA expression levels of NAMPT, ZFP36, CASP1, CTSD, HMOX1, and PYCARD were elevated in AS and PsD cell models (**Figure 10**). Western Blot (WB) results indicated that the expression levels of NAMPT, ZFP36, CASP1, CTSD, HMOX1, and PYCARD were significantly increased in AS and PsD cell models (**Figure 11**). This further validates the conclusions drawn in this study.

### 3.6 Investigating immune cell infiltration and its correlation with key genes

**Figure 12:**
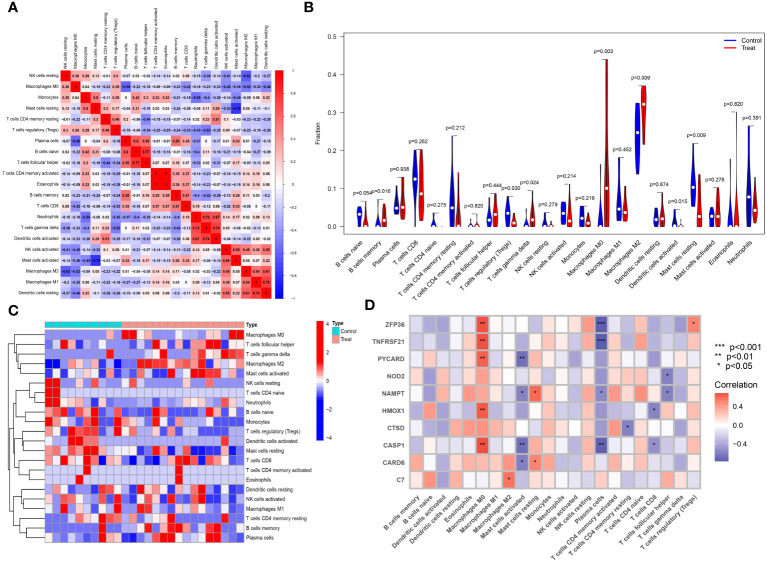
Immune cell infiltration composition in AS. **(A)** Heatmap of immune cell correlations in AS. **(B)** Violin plot of immune cell infiltration differences between early atherosclerotic plaques and advanced atherosclerotic plaques samples. **(C)** Heatmap of the proportion of various immune cells in early atherosclerotic plaques compared to advanced atherosclerotic plaques. **(D)** Heatmap showing correlations between immune cells and key genes.

**Figure 13:**
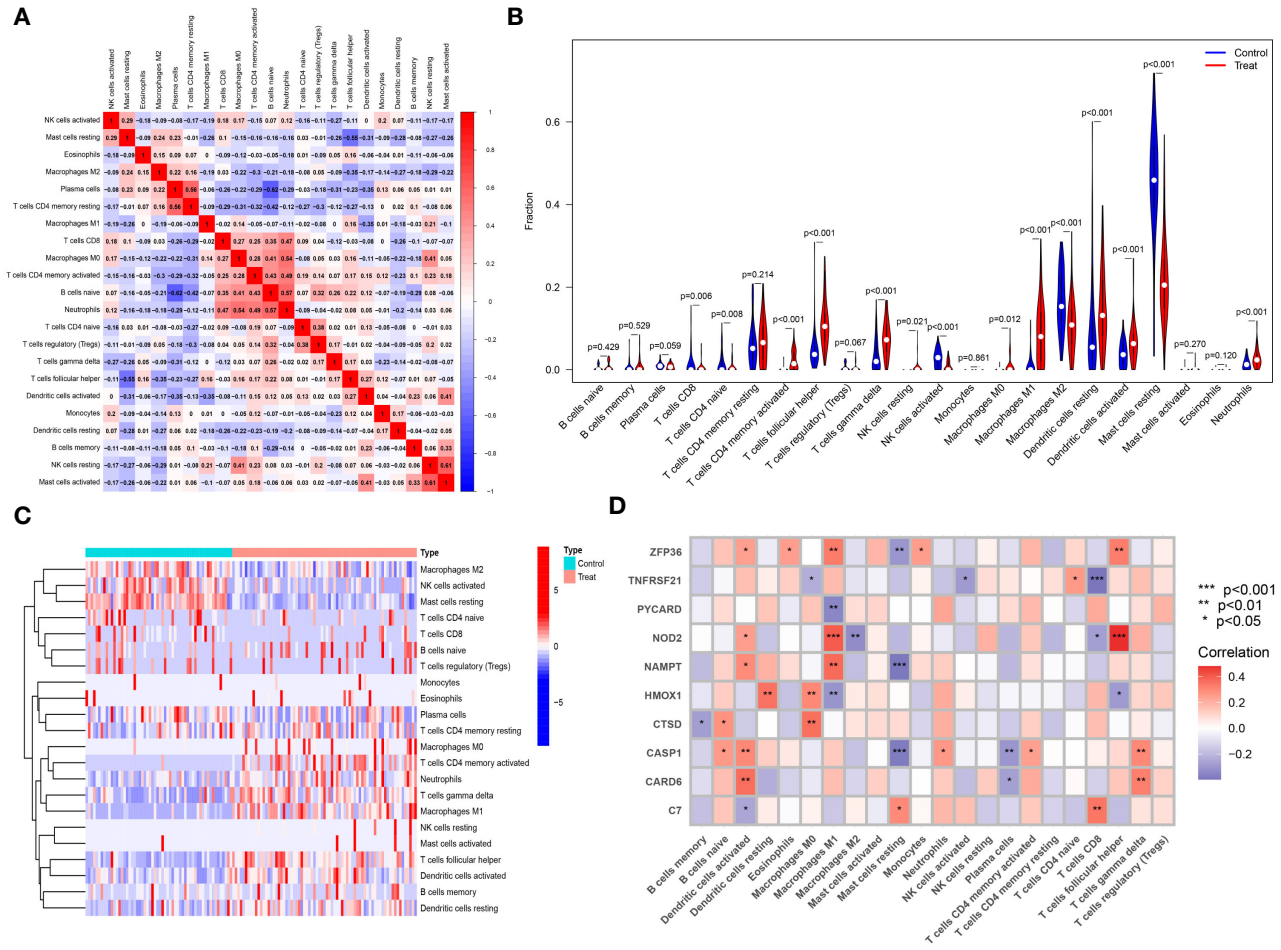
Immune cell infiltration composition in PsD. **(A)** Heatmap of immune cell correlations in PsD. **(B)** Violin plot of immune cell infiltration differences between skin lesions (LS) and adjacent normal tissues (NL). **(C)** Heatmap of the proportion of various immune cells in skin lesions (LS) and adjacent normal tissues (NL). **(D)** Heatmap showing correlations between immune cells and key genes.

The CIBERSORT algorithm was employed to analyze differences in the immune microenvironment between patients and normal samples in the GSE28829 and GSE30999 datasets. In the GSE28829 dataset, as illustrated in **Figures 12A**, **13A**, a significant negative correlation was found between activated Mast cells and resting Mast cells (r=-0.76), while follicular helper T cells showed a significant positive correlation with naive B cells (r=0.77). In the GSE30999 dataset, naive B cells had a significant positive correlation with Plasma cells (r=-0.62), and activated Mast cells were significantly positively correlated with resting NK cells (r=0.61). Compared to normal samples, higher levels of memory B cells, gamma delta T cells, Macrophages M0, and Macrophages M2 were detected in AS samples (**Figure 12B**). In PsD samples, increased levels of CD4 memory activated T cells, gamma delta T cells, Macrophages M0, M1, M2, activated Dendritic cells, and Neutrophils were observed (**Figure 13B**). Heatmaps depicted the proportions and differences in 22 types of immune cell infiltrations between AS, PsD, and normal samples (**Figures 12C**, **13C**). Furthermore, Spearman correlation analysis showed that in AS samples, ZFP36, TNFRSF21, PYCARD, HMOX1, and CASP1 were significantly positively correlated with Macrophages M0, while ZFP36, TNFRSF21, and CASP1 exhibited a significant negative correlation with Plasma cells (**Figure 12D**). In PsD samples, ZFP36, NOD2, NAMPT were significantly positively correlated with Macrophages M1, and NAMPT and CASP1 were significantly negatively correlated with resting Mast cells (**Figure 13D**). These results indicate a close relationship between changes in the immune microenvironment of AS and PsD patients and the expression of feature genes such as ZFP36, TNFRSF21, PYCARD, HMOX1, and CASP1.

### 3.7 Prediction of small molecule compounds targeting key genes for the treatment of atherosclerosis and psoriasis

**Figure 14:**
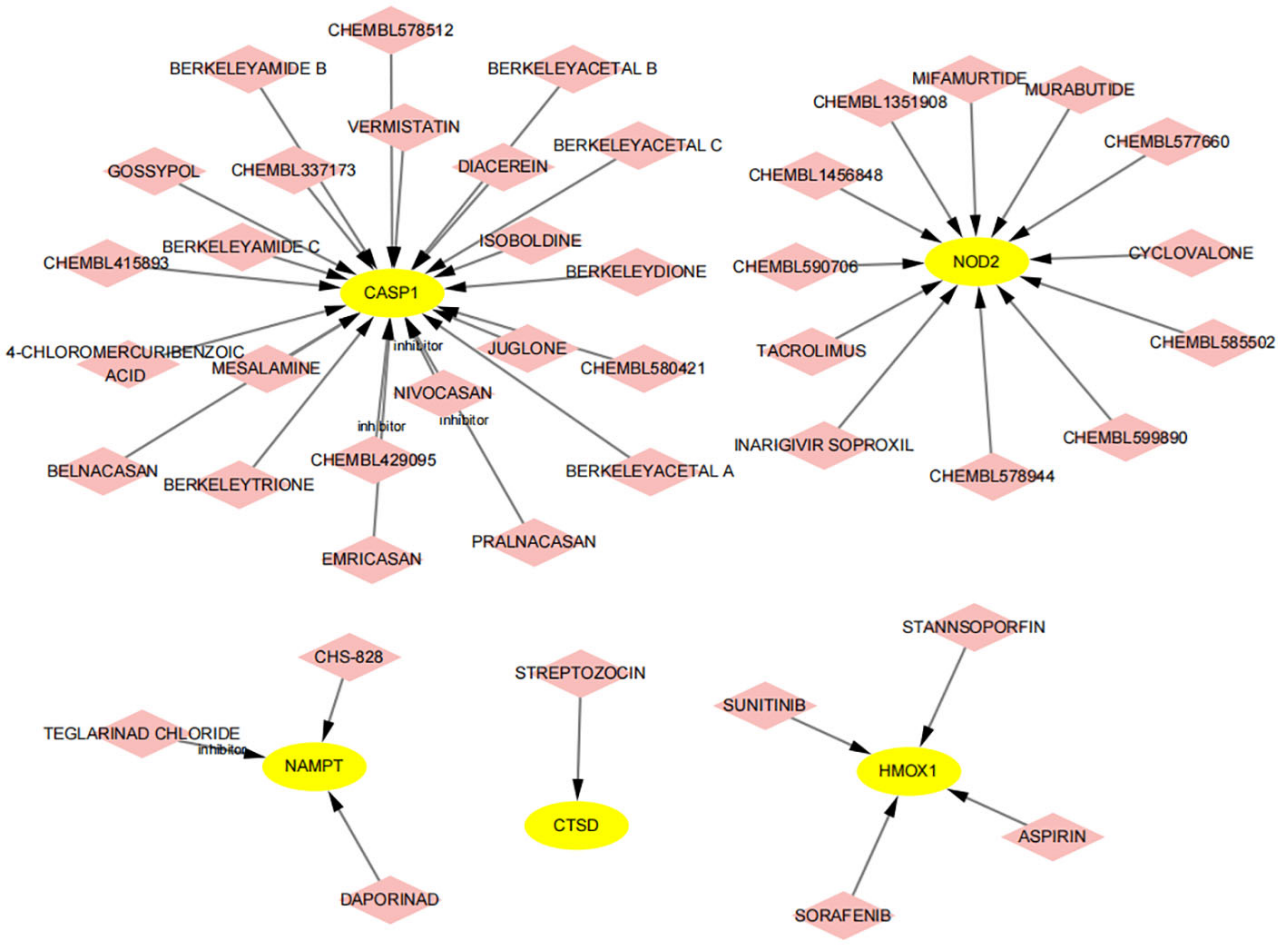
Prediction of drugs targeting feature genes. Yellow indicates feature genes, and pink represents drugs.

Using the DGIdb database, we analyzed potential therapeutic drugs that could counteract abnormal mean gene expression. This study predicted drugs that might target the previously identified feature genes. Through DGIdb, 43 drugs targeting these genes were identified (**Figure 14**), including 23 drugs or molecular compounds such as NIVOCASAN, EMRICASAN, PRALNACASAN, BERKELEYAMIDE C, and CHEMBL337173, which interact with CASP1. Additionally, 12 drugs or compounds, including MIFAMURTIDE, MURABUTIDE, and CHEMBL1456848, were noted for their potential to modulate NAMPT expression. Drugs such as SORAFENIB, SUNITINIB, STANNSOPORFIN, and ASPIRIN were identified as potential targets for HMOX1. Regrettably, no drugs were found that could regulate the expression of ZFP36, C7, CARD6, PYCARD, and TNFRSF21, highlighting the need for further research in this area.

### 3.8 Constructing a ceRNA network based on key genes

**Figure 15:**
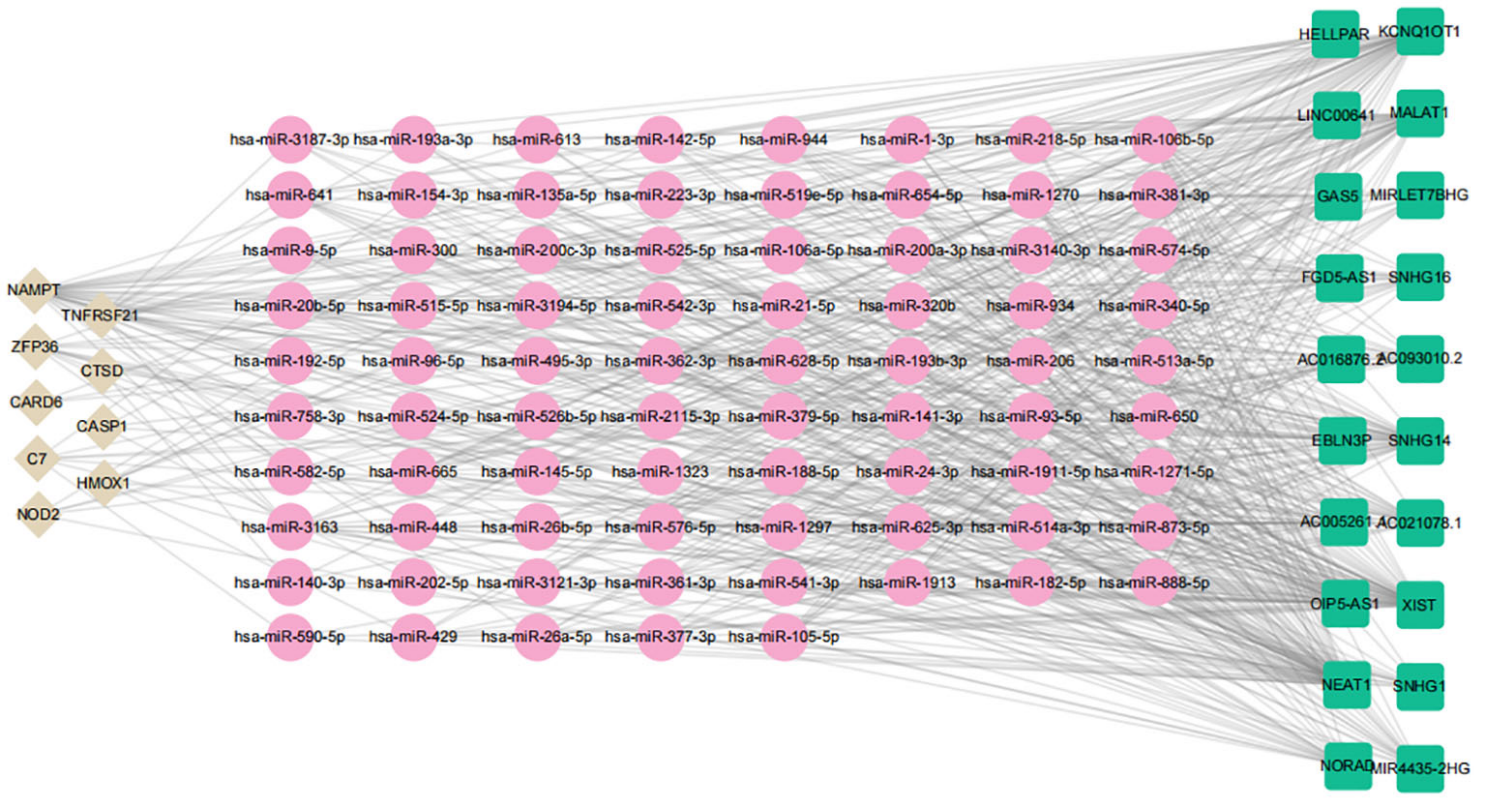
ceRNA regulatory network of hub genes. Brown diamonds represent hub genes; pink circles denote miRNAs, and green rectangles indicate lncRNAs.

Emerging research indicates that lncRNAs can bind competitively to target miRNAs through complementary base pairing, influencing gene expression in what is known as the ceRNA network (38). Based on this concept, we constructed a ceRNA network that includes 2 DE-FRGs and 7 DE-NRGs. The network consists of 106 nodes (9 hub genes, 76 miRNAs, and 21 lncRNAs) and 592 edges (**Figure 15**). According to the ceRNA hypothesis, literature review revealed that miR-142–5p is significantly expressed in the aortic plaques of AS patients and ApoE^−/−^ mice, as well as in the skin of subjects with allergic reactions. Overexpression of miR-142–5p inhibits the expression of NAMPT. Additionally, studies have indicated that NEAT1 and XIST have putative binding sites for miR-142–5p, supporting that miR-142–5p is a direct target of NEAT1 and XIST (39, 40). Similarly, miR-192–5p is significantly elevated in the serum of patients with AS and psoriasis, and its overexpression suppresses NOD2 expression. Further research has identified specific binding sites of KCNQ1OT1 with miR-192–5p, suggesting that miR-192–5p is a direct target of KCNQ1OT1 (41–43). Consequently, we selected hsa-miR-142–5p and hsa-miR-192–5p to construct subnetworks for further analysis (**Figure 15**). By integrating the regulatory relationships between lncRNA, miRNA, and mRNA, the NEAT1/XIST-142–5p-NAMPT and KCNQ1OT1-miR-192–5p-NOD2 pathways may represent critical pathways in the pathogenesis of AS and PsD.

## 4 Discussion

PsD and AS are prevalent diseases in clinical settings, causing significant economic losses and societal burdens worldwide (44, 45). Prior research has established a close association between PsD and systemic chronic inflammation, which also plays a critical role in AS and its complications, such as myocardial infarction and stroke (46). Chronic inflammation in PsD patients can lead to insulin resistance and endothelial cell dysfunction, thereby exacerbating AS and various cardiovascular diseases (47). Studies have demonstrated an increased risk of cardiovascular diseases in PsD patients, a risk that escalates with the severity and duration of the condition (48). Moreover, genes related to iron death and necroptotic apoptosis are implicated in the progression of both PsD and AS. However, the interplay between PsD, AS, and these apoptotic processes remains poorly understood (49–52). Consequently, our study aims to explore the shared characteristics and molecular pathways of iron death and necroptotic apoptosis genes in PsD and AS from a bioinformatics perspective, utilizing existing sequencing databases to offer novel insights into the pathogenesis of these diseases.

In this research, we identified 44 DE-FRGs and 30 DE-NRGs and conducted functional enrichment analysis. The 44 DE-FRGs were primarily enriched in pathways such as Ferroptosis, NOD-like receptor signaling, TNF signaling, PPAR signaling, and IL-17 signaling. Conversely, the 30 DE-NRGs exhibited significant enrichment in pathways such as Necroptosis, NOD-like receptor signaling, TNF signaling, and the p53 signaling pathway. These findings highlight the pivotal role of inflammation in both PsD and AS. Importantly, the NOD-like receptor signaling pathway and TNF signaling pathway were significantly prevalent in both diseases, underscoring their fundamental roles in the pathogenesis of these conditions. NOD-like receptors (NLRs), belonging to the cytoplasmic pattern recognition receptor family, are crucial to the innate immune system and are closely linked with the pathogenesis of AS (53). For example, a study found that two ferroptosis-related proteins, PTGS2 and ACSL4, and two necroptosis-related proteins, NLRP3 and caspase-1, are upregulated in the advanced stages of atherosclerosis (AS) and are positively correlated with the severity of AS. These findings support the credibility of the enriched results for ferroptosis and necroptosis in this study, suggesting that proteins involved in these processes may be potential targets for regulating AS, and their expression levels could serve as indicators of AS severity (54). Furthermore, compared to healthy individuals, NLRP3 mRNA is upregulated in the plaques and psoriatic dermatitis (PsD) epidermis of AS patients. Recent studies indicate that activation of the NLRP3 inflammasome can induce ferroptosis, highlighting that inhibiting NLRP3 activation may suppress the occurrence of ferroptosis in AS and PsD (55). Tranexamic acid (TXA) inhibits IL-17-induced ROS production, thereby suppressing IL-17-induced activation of NFκB, expression of the NLRP3 inflammasome, and Nrf2-mediated expression of keratin 17, thus exerting an anti-PsD effect (56). Moreover, NLRP3 expression levels in PsD samples are 3.5 to 4.3 times higher than in normal skin biopsy samples, indirectly indicating that inflammasome activation is closely associated with the pathogenesis of PsD (57). Additionally, TNF, a pro-inflammatory cytokine produced by activated leukocytes, targets endothelial cells and triggers a cascade of events, leading to endothelial dysfunction and accelerated development of AS (58). TNF also regulates the NF-κB signaling pathway, activates inflammatory genes, and promotes the expression of cell adhesion molecules, indirectly inducing endothelial cell apoptosis and necroptosis, thereby accelerating the progression of AS (59). The TNF signaling pathway is markedly upregulated in imiquimod-induced mouse psoriasis lesions, and TNF-α, a crucial target of this pathway, mediates inflammatory responses and is an inflammatory cytokine linked to PsD pathogenesis. Anti-TNF-α therapy has shown particular effectiveness in PsD treatment (60). These evidences show that ferroptosis-related genes and necroptosis-related genes related to AS and PsD are mainly enriched in immune and inflammatory signaling pathways, suggesting that immune-inflammatory signaling pathways mediated byferroptosis and necroptosis may be the potential mechanisms of AS and PsD.

Subsequently, we selected feature genes using three machine learning algorithms, focusing on 2 ferroptosis-related genes (NAMPT, ZFP36) and 8 necroptosis-related genes (C7, CARD6, CASP1, CTSD, HMOX1, NOD2, PYCARD, TNFRSF21). NAMPT, an enzyme involved in NAD biosynthesis, is highly expressed in the serum of neck AS patients, with significantly elevated plasma eNAMPT levels in ApoE knockout AS mice (61, 62). Additionally, eNAMPT may contribute to AS development by inducing endothelial dysfunction (63). Similarly, the NAMPT-mediated NAD salvage pathway enhances the epithelial autocrine inflammatory response in PsD, thus promoting its pathogenesis (64). This suggests that NAMPT-mediated inflammation may play a role in the development of both AS and PsD. ZFP36, minimally expressed in healthy aortas but significantly in endothelial cells and macrophages in mouse and human AS lesions, inhibits pro-inflammatory mRNA transcripts, thereby reducing vascular inflammation (65). Furthermore, ZFP36 family protein expression is diminished under chronic inflammatory conditions similar to those in PsD lesions, contributing to the onset of PsD’s inflammatory phenotype (66). Additionally, the human ZFP36 family comprises three members (ZFP36, ZFP36L1, and ZFP36L2), among which ZFP36 plays distinct roles compared to the other two. Different family members bind to varied DNA binding sites and regulate different molecular pathways. In other words, even if expressed at the same levels, ZFP36 may exert unique functions in transcriptional activity. The alternative splicing of ZFP36 could explain its higher expression levels in the PsD research cohort and lower levels in the validation cohort (65). HMOX1, highly expressed in the endothelium of AS mice, plays a role in reducing iron death in AS endothelial cells when knocked out, thus mitigating Fe^2+^ overload and lipid peroxidation (67). Elevated expression of HMOX1 in the skin of PsD patients is consistent with our findings (68), suggesting that inhibiting HMOX1 expression might be a shared target for AS and PsD treatment. CASP1, part of the inflammasome, is expressed at low levels in ApoE knockout mice, leading to decreased vascular inflammation and AS progression (69). Clinically, CASP1 expression is elevated in PsD patients, supporting our results (70). NOD2, pivotal in integrating endoplasmic reticulum stress and inflammation, is essential for endothelial cell apoptosis and AS progression (71). Previous research suggests that NOD2 deficiency leads to reduced lipid deposition and diminished inflammatory cell infiltration in AS plaques (72). Correspondingly, the observation of upregulated NOD2 in PsD epidermis supports these findings (73). Additionally, research data indicate that abnormalities in complement components and the resultant overactivation are associated with the formation of AS. Studies have found higher levels of complement component C7 in intimal thickening and fibrous plaques compared to normal tissues (74). However, another study confirmed that increased concentrations of C7, which is involved in the formation of the membrane attack complex, were not observed in the blood of patients with unstable plaques. This might be due to the process occurring locally within AS lesions, indirectly explaining why C7 exhibits higher expression in the AS research cohort and lower expression in the validation cohort (75). However, the roles of CARD6, CTSD, PYCARD, and TNFRSF21 in PsD and AS remain underexplored, emphasizing their significance in future research and the necessity to further investigate their specific mechanisms.

To further investigate the role of immune cells in PsD and AS, an extensive assessment of immune cell infiltration in these diseases was conducted using CIBERSORT. The findings indicated that in AS samples, memory B cells, gamma delta T cells, Macrophages M0, and Macrophages M2 were markedly present. Conversely, in PsD samples, an increase was noted in CD4 memory activated T cells, gamma delta T cells, Macrophages M0, M1, M2, activated Dendritic cells, and Neutrophils. The significant infiltration of gamma delta T cells and Macrophages in both PsD and AS led to an examination of their shared pathogenic mechanisms in these diseases. Recent studies have identified IL-23R^+^ gamma delta T cells to be prevalent in the aortic root of Ldlr^-/-^ Il23rgfp/^+^ mice. It was found that the absence of specific gamma delta T cell subsets reduced aortic root AS lesions, and high-throughput sequencing data confirmed that gamma delta T cells predominantly express IL-23R and IL-17A in the aorta (76). Previous research has identified dermal gamma delta T cells as key sources of IL-17A, IL-22, and IL-17F, playing a crucial role in the initiation and development of PsD (77, 78). Furthermore, reports suggest that inhibiting the production of IL-17A by gamma delta T cells effectively reduces inflammatory symptoms in PsD skin tissue (79). Therefore, gamma delta T cells and their secreted cytokines are fundamental to the pathogenesis of both PsD and AS. Macrophages, vital to immune cell function in AS, efficiently remove necrotic and dead cells from lesions, thus alleviating tissue damage and the progression of AS (80). PsD patient epidermal tissues show a significant increase in macrophages, linked with hyperproliferation and inflammation. The persistence of skin inflammation in PsD is heavily dependent on macrophage recruitment and activation and the release of TNF-α (81, 82). In summary, the development of PsD and AS is intimately connected to the extent of immune cell infiltration, and understanding the underlying mechanisms of these immune cells is vital for the prevention and treatment of PsD and AS.

Furthermore, we sourced drug-gene interaction data from the DGIdb database, identifying a total of 43 potential drugs or compounds for treating PsD and AS. DIACEREIN, an anthraquinone derivative known for its anti-inflammatory, antipyretic, and analgesic properties, is commonly used in osteoarthritis treatment (83). Recent research indicates that DIACEREIN mitigates IL-1-induced skin inflammation in PsD and curbs the development of IL-1-induced AS (84). JUGLONE, a natural phenolic compound derived from walnut tree roots, stems, and leaves, has shown significant efficacy in alleviating endothelial dysfunction, oxidative stress, and NF-kB-driven inflammatory responses in diabetic mice, thus potentially preventing cardiovascular complications of diabetes (85). However, current literature does not document the use of JUGLONE in PsD treatment, emphasizing the need for further exploration of its application in PsD. Tacrolimus, extensively used as an immunosuppressant in various organ transplants, exhibits anti-inflammatory properties. Recent studies suggest that Tacrolimus may inhibit AS formation by curtailing ROS and NLRP3 inflammasome activation in macrophages, along with reducing IL-1β and IL-18 release (86). Tacrolimus is also recommended for PsD treatment, particularly in areas with thinner skin. The latest research indicates that microneedle patches laden with Tacrolimus nanocrystals can effectively ameliorate histopathological features of PsD skin and decrease TNF-α, IL-17A, and IL-23 levels (87). This study suggests that immune disorder-mediated inflammatory responses are instrumental in the onset of PsD and AS, indicating immunotherapy as a promising treatment approach for both conditions. While drug repurposing is a viable strategy for identifying therapeutic candidates, these proposed drugs and target genes require further validation through extensive animal studies and clinical trials.

To delve deeper into the mechanisms of 2 ferroptosis-related genes (NAMPT, ZFP36) and 8 necroptosis-related genes (C7, CARD6, CASP1, CTSD, HMOX1, NOD2, PYCARD, TNFRSF21) in AS and PsD, we developed a ceRNA network relevant to both conditions. This network highlighted NEAT1, XIST, KCNQ1OT as key lncRNAs, hsa-miR-3163, hsa-miR-93–5p, and hsa-miR-20b-5p as essential miRNAs, and ZFP36 and HMOX1 as critical mRNAs. NEAT1 exhibited increased expression in AS patients and ox-LDL-induced HAECs, and its downregulation significantly reduced the proliferation of ox-LDL-activated HAEC cells while promoting apoptosis (88). Similarly, XIST was upregulated in the serum of AS patients and Ox-LDL-induced VSMCs, with XIST inhibition leading to reduced cell vitality and increased apoptosis (89). Furthermore, miR-93–5p overexpression inhibited endothelial cell ferroptosis, and as previously mentioned, both ZFP36 and HMOX1 were upregulated in AS models (90). We postulate that XIST and NEAT1 may function as miR-93–5p sponges, impacting ZFP36 and HMOX1 expression and thereby contributing to AS progression. However, the regulatory influence of NEAT1, XIST, and KCNQ1OT on miRNAs in PsD remains unexplored, emphasizing the need for future research focusing on additional ncRNAs in PsD mechanisms.

In summary, we identified shared DE-FRGs and DE-NRGs in AS and PsD and performed enrichment analysis. Our findings revealed numerous common pathogenic mechanisms between AS and PsD, potentially mediated by specific hub genes. Using three machine learning algorithms, we pinpointed 2 ferroptosis-related genes (NAMPT, ZFP36) and 8 necroptosis-related genes (C7, CARD6, CASP1, CTSD, HMOX1, NOD2, PYCARD, TNFRSF21) as key biomarkers, which demonstrate positive or negative correlations with certain immune cells, potentially affecting the immune microenvironment in AS and PsD. Further investigation into these immune cells may assist in developing immunoregulatory treatments for AS and PsD. However, this study has certain limitations. Firstly, a major weakness is the insufficient sample size; in the future, we could incorporate additional datasets to expand the sample pool. Secondly, there might be bias in the selection of training and validation datasets. Although this study utilized publicly available datasets, they may not represent the broader AS or PsD patient populations. Thirdly, due to budget constraints, this study did not further explore and validate mechanisms and pathways between target organs, novel DEGs, and immune cells. Including external validation cohorts and conducting multifunctional experiments is crucial for our research. The former would enhance the robustness and generalizability of our findings, while the latter would provide deeper insights into unknown molecular mechanisms. We recommend future multidisciplinary collaborations and further basic experiments. Lastly, although our study validated the functions of NAMPT, ZFP36, CASP1, CTSD, HMOX1, and PYCARD through RT-qPCR and WB, the precise molecular regulatory mechanisms still require further investigation. More clinical samples are needed to confirm our findings. Moreover, the regulatory mechanisms of necroptosis and ferroptosis are highly complex. Although we identified NAMPT and ZFP36 as ferroptosis-related genes that may be associated with necroptosis-related genes such as CASP1, CTSD, HMOX1, and PYCARD, how they interact to influence the progression of AS and PsD warrants deeper study. We hope to construct stable cell lines with knockdown and overexpression of these genes in subsequent research to explore and validate the results obtained. Nonetheless, this study is limited as our conclusions are based solely on data analysis and lack experimental validation. Therefore, future animal studies are necessary to validate our findings. Despite these limitations, our research offers novel insights into the comorbidity of AS and PsD, providing new perspectives for exploring ferroptosis-related genes and necroptosis-related genes and molecular mechanisms in these diseases.

## Data availability statement

The datasets presented in this study can be found in online repositories. The names of the repository/repositories and accession number(s) can be found in the article/supplementary material.

## Ethics statement

The manuscript presents research on animals that do not require ethical approval for their study.

## Author contributions

JF: Conceptualization, Data curation, Formal analysis, Methodology, Software, Validation, Writing – original draft. TZ: Project administration, Software, Writing – review & editing. XT: Methodology, Supervision, Writing – review & editing. SL: Investigation, Methodology, Supervision, Writing – original draft. S-LZ: Project administration, Writing – review & editing.
